# Prospective Study of a Cohort of Russian Nijmegen Breakage Syndrome Patients Demonstrating Predictive Value of Low Kappa-Deleting Recombination Excision Circle (KREC) Numbers and Beneficial Effect of Hematopoietic Stem Cell Transplantation (HSCT)

**DOI:** 10.3389/fimmu.2017.00807

**Published:** 2017-07-24

**Authors:** Elena Deripapa, Dmitry Balashov, Yulia Rodina, Alexandra Laberko, Natalya Myakova, Nataliia V. Davydova, Maria A. Gordukova, Dmitrii S. Abramov, Galina V. Pay, Larisa Shelikhova, Andrey P. Prodeus, Mikhail A. Maschan, Alexey A. Maschan, Anna Shcherbina

**Affiliations:** ^1^Dmitry Rogachev National Research and Clinical Center for Pediatric Hematology, Oncology and Immunology, Moscow, Russia; ^2^Speransky Children’s Hospital, Moscow, Russia

**Keywords:** Nijmegen breakage syndrome, hematopoietic stem cell transplantation, T-cell excision circle, kappa-deleting recombination excision circle, malignancy, granuloma

## Abstract

**Background:**

Nijmegen breakage syndrome (NBS) is a combined primary immunodeficiency with DNA repair defect, microcephaly, and other phenotypical features. It predominantly occurs in Slavic populations that have a high frequency of carriers with the causative NBN gene c.657_661del5 mutation. Due to the rarity of the disease in the rest of the world, studies of NBS patients are few. Here, we report a prospective study of a cohort of Russian NBS patients.

**Methods:**

35 Russian NBS patients of ages 1–19 years, referred to our Center between years 2012 and 2016, were prospectively studied.

**Results:**

Despite the fact that in 80% of the patients microcephaly was diagnosed at birth or shortly thereafter, the average delay of NBS diagnosis was 6.5 years. Though 80% of the patients had laboratory signs of immunodeficiency, only 51% of the patients experienced significant infections. Autoimmune complications including interstitial lymphocytic lung disease and skin granulomas were noted in 34%, malignancies—in 57% of the patients. T-cell excision circle (TREC)/kappa-deleting recombination excision circle (KREC) levels were low in the majority of patients studied. Lower KREC levels correlated with autoimmune and oncological complications. Fifteen patients underwent hematopoietic stem cell transplantation (HSCT), 10 of them were alive and well, with good graft function. Three patients in the HSCT group and five non-transplanted patients died; tumor progression being the main cause of death. The probability of the overall survival since NBS diagnosis was 0.76 in the HSCT group and 0.3 in the non-transplanted group.

**Conclusion:**

Based on our findings of low TRECs in most NBS patients, independent of their age, TREC detection can be potentially useful for detection of NBS patients during neonatal screening. KREC concentration can be used as a prognostic marker of disease severity. HSCT is a viable treatment option in NBS and should be especially considered in patients with low KREC numbers early on, before development of life-threatening complications.

## Introduction

Nijmegen breakage syndrome (NBS) is a combined primary immunodeficiency, additionally characterized by a DNA repair defect and syndromic features (1). NBS is caused by recessively inherited mutations of NBN (formerly NBS1) gene (2), which encodes nibrin protein—a member of the Mre11/Rad50/NBN multiprotein nuclear complex involved in detection and correction of physiological and pathological double-stranded DNA breaks (3). NBS has variable prevalence in various parts of the world, with highest being in Slavic countries (Poland, Russia, Ukraine, etc.). Several truncating mutations on NBN have been described, c.657_661del5 in exon 6 being the most frequent due to the founder effect in Slavic population, in which carrier frequency as high as 1:154 has been reported (4).

Syndromic features in NBS include microcephaly presenting at birth (or even *in utero*), bird-like facial features, variable growth delay and mental retardation, hyper- or hypopigmented skin lesions, various organ malformations (poly-syndactyly, kidney anomalies, hypospadias, cryptorchidism) [summarized in Ref. (5)].

Immune defects in NBS vary in severity and affect both humoral (low B cells, hypoagammaglobulinemia) and cellular (low T cells, low naïve T cells) compartments (6). It has been demonstrated that at least some NBS patients can be detected *via* neonatal T-cell excision circles (TRECs) screening (7). Also, low kappa-deleting recombination excision circle (KREC) numbers have been reported in NBS (8). Yet, T-cell excision circle (TREC)/KREC data in NBS are scarce and require further investigation.

Symptoms of the disease include bacterial and viral infections, autoimmune complications, including skin sarcoid-like granulomas. Yet, the most prominent feature of the disease is predisposition to malignancies. In opposite to other primary immunodeficiencies not only lymphoreticular but also an array of solid tumors have been reported in NBS. There is no consensus on tumor treatment protocols for NBS, though most centers omit or reduce doses of alkylating agents (according to individual tolerability) and avoid irradiation (9).

Hematopoietic stem cell transplantation (HSCT) in NBS is controversial, since there is no evidence it reduces the risk of malignancies. If HSCT is attempted, reduced conditioning regiments are usually used (10).

Here, we report clinical and laboratory features of 35 NBS patients referred to our Center between years 2012 and 2016.

## Materials and Methods

### Patients

Between 2012 and 2017, 35 patients from 33 unrelated families with preliminary diagnosis of NBS have been referred to our Center. Included were seven previously described patients, with updated clinical information. There were 16 males and 19 females, median age at referral was 13.4 years (range 1–19 years). The diagnosis of NBS was based on ESID diagnostic criteria for NBS and was confirmed in all patients by detection of homozygous deletion 657_661 delACAAA in the NBN gene.

### Clinical Analysis

Clinical symptoms of each patient were analyzed retrospectively from birth and prospectively during the follow-up. Types, severity and frequency of infections, autoimmune complications and malignancies, treatment protocols, and outcomes were analyzed.

Infectious complications were qualified in two categories according to their severity. Severe infections were defined as more than two episodes of pneumonia per year, any infection requiring intravenous antibiotics use, oxygen or intravenous fluids supplementation and\or clearly linked to death within two weeks.

### Laboratory Analysis

B and T lymphocytes subsets were analyzed by standard flow cytometry methods. Patients who had less than 1,000 T cells and less than 200 B cells were reported. IgG, IgM, and IgA levels were assayed by standard nephelometry assay. Patients who had significant decrease of one or more immunoglobulin classes (by 2 or more SDs from mean) were reported.

### Rubella PCR Detection

Rubella virus was assayed by standard PCR method in DNA extracted from granulomatous skin lesions’ biopsies using «AmliSense^®^ Rubella virus-FL» kit (InterLabservice, Russia) according to manufacturer’s instructions.

### TREC/KREC Detection

The TREC/KREC levels were assayed in whole blood samples as described previously (11). Briefly, DNA was extracted from 100 µl EDTA anticoagulated whole blood by using RIBO-prep nucleic acid extraction kit (Amplisense^®^, Russia).

The Real-time qPCR for TRECs and KRECs was performed by using T&B PCR kit (ABV-test, Russia) on CFX 96 Real-Time PCR System (Bio Rad, USA). Amplification of ALB was used to assess correct sampling and quality of DNA extraction and to determine of TRECs and KRECs levels. The number of copies of TRECs (KRECs) was calculated per 10^5^ white blood cells, taking into account the quantity of ALB using formula: [The number of copies TRECs (KRECs)/the number of copies ALB] × 200,000.

The normal/cutoff levels of TRECs and KRECs of 1,000 copies/10^5^ cells were used.

### Hematopoietic Stem Cell Transplantation

Nijmegen breakage syndrome patients underwent HSCT in accordance with institutional protocol (12). The source of stem cells was bone marrow (*n* = 2) or peripheral blood (*n* = 13) of matched related (*n* = 4) or unrelated (*n* = 11) donors. All peripheral blood stem cells were TCRαβ/CD19 depleted *via* immunomagnetic method in accordance with manufacture’s instructions (Miltenyi Biotec, Bergisch Gladbach, Germany). Conditioning consisted of Busulfan (4 mg/kg), Fludarabine (150 mg/m^2^), Cyclophosphamide (40 mg/kg, *n* = 12; 30 mg/kg, *n* = 1; 20 mg/kg, *n* = 1), and ATG (rabbit) (5 mg/kg) (total dose given over several days). On day-1, most patients (14 out of 15) received low-dose rituximab 100 mg/m^2^ in order to reduce the risk of Epstein–Barr virus-related post-transplantation lymphoproliferative disease. GVHD prophylaxis consisted of tacrolimus in 10 cases, tacrolimus, and a short course of methotrexate in 5 cases.

### Statistical Analysis

Demographic, disease-related, or transplant-related characteristics were described with frequencies for categorical variables, and median and range for quantitative variables. Probability of overall survival was estimated by the Kaplan–Meier product limit and were expressed as percentage ± SD. Patients were censored at the time of death, survivors on March 30, 2017. The two-sided log-rank test was used for comparisons. For the comparison of TREC and KREC levels, the Mann–Whitney test was used. For the analysis of correlation between TREC and KREC levels and age, the linear regression method was used. The time of NBS diagnosis was chosen as a starting point for survival estimation, since it is the time when the decision about overall treatment strategy (i.e., HSCT vs not HSCT) is made. TREC and KREC levels were expressed as mean ± SEM. *p-*values less than 0.1 were considered statistically significant. Statistical analysis was performed using XLSTAT, Addinsoft, 2015 software.

## Results

### Demographics and Non-Oncological Clinical Manifestations

In our cohort of 35 patients, 32 considered themselves Russians, two Ukranians, and one Gypsy. In three patients, microcephaly and/or syndromic features were noted during prenatal ultrasound, in 20—at birth. Five patients were followed by neurologist due to microcephaly and cognitive impairment since the first year of life. Yet, in 15 patients, the diagnosis of NBS was made only by hematologist/oncologists at the time of malignant complication. The median time of NBS diagnosis was 5.0 years (1–16 years), the average diagnosis delay was 6.5 years. All patients received intravenous immunoglobulin (IVIG) therapy upon NBS diagnosis irrespective of immunoglobulin levels.

Seventeen patients (49%) had mild to moderate infectious episodes, 18 (51) patients suffered from severe recurrent bacterial infections, which were the cause of death in three patients (Table 1).

**Table 1:** Infectious, autoimmune, and oncological complications in 35 patients with Nijmegen breakage syndrome.

Symptoms	Number of patients	Age of manifestation, years, median (min–max)	Cause of death
**Infections**			
No/mild	17	5.8 (1–11)	
Severe	18	7.1 (2–17)	3
**Autoimmune complications**			
Arthritis	1	14	
Thyroiditis	2	10 (6–14)	
Skin granulomas	5	9.4 (4–13)	
Interstitial lymphocytic lung disease	4	9.75 (6–13)	
**Malignancies**			
B cell lymphoma	10	8.7 (5–13)	
T cell lymphoma	4	9.5 (4–18)	3
Acute lymphoblastic leukemia	7	7.0 (2–12)	1
T-prolymphocytic leukemia	1	6	
Ganglioglioma	1	15	1
**Other**			
Lymphomatoid granulomatosis	3	13 (8–17)	

**Figure 1:**
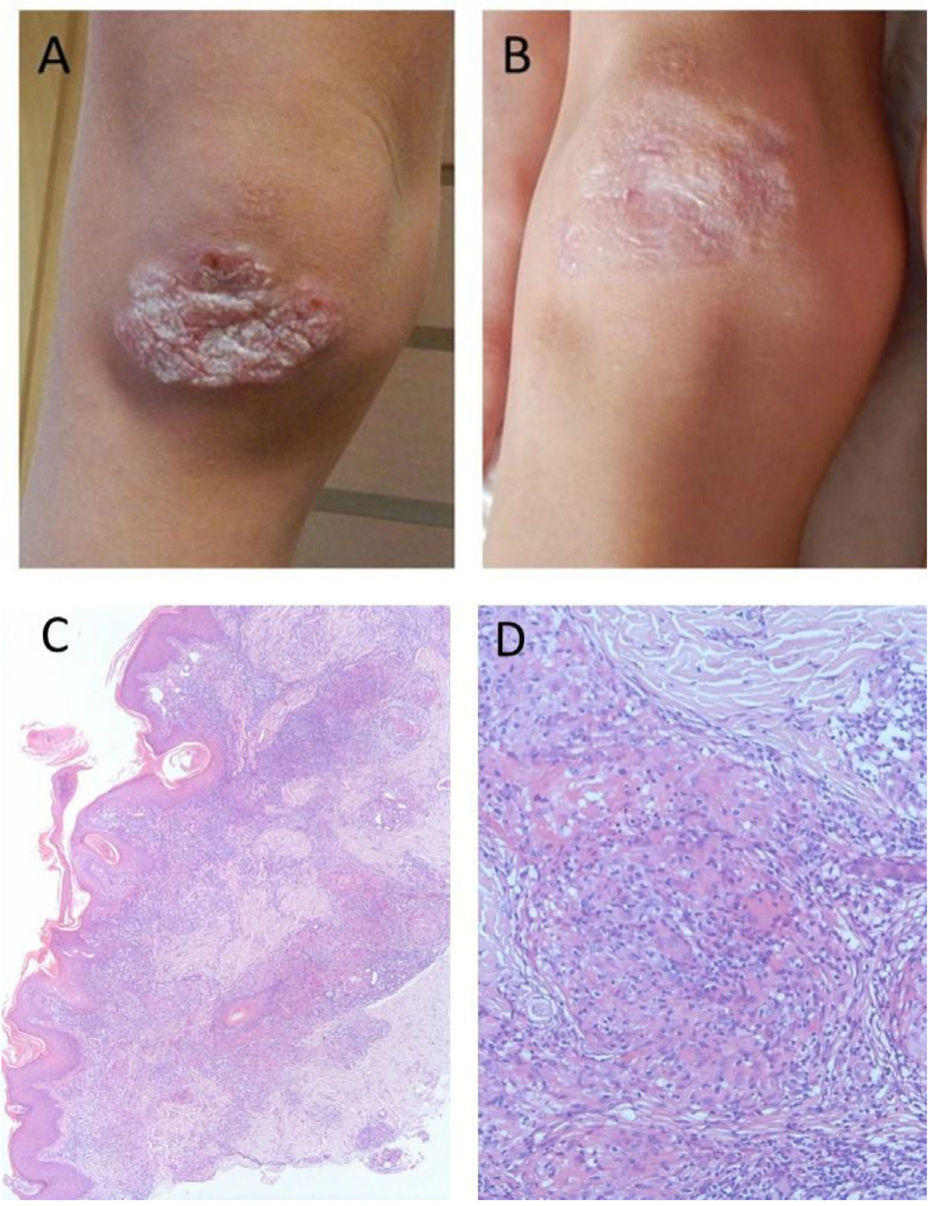
Macroscopic and microscopic images of granulomatous lesion in a boy with Nijmegen Breakage Syndrome: **(A)** at referral, after multiple courses of antibiotics and topical steroids, **(B)** after conditioning for hematopoietic stem cell transplantation, **(C)** skin histology demonstrating perivascular and diffuse, mixed-cell infiltrate in superficial and deep dermis. H&E stain, 40× magnification, **(D)** skin histology demonstrating palisaded inflammatory infiltrates consisting of histocytes, lymphocytes, and neutrophils, associated with basophilic alterations of collagen. H&E stain, 400× magnification.

Twelve patients (34%) developed autoimmune complications with median age of onset 10.5 years (4–14 years) (Table 1). Autoimmune complications included arthritis in one, thyroiditis in two, interstitial lymphocytic lung disease (ILLD) in four, skin granulomas in five (Figure 1A). Skin biopsies were performed in all cases of granulomatous skin lesions (median age of manifestation 9.4 years). Upon biopsy, all of them demonstrated signs of epithelioid granulomas with foci of necrosis (Figures 1C,D). Retrospectively, in three out of five skin granuloma biopsies, Rubella virus was detected by PCR. Precluding treatment of skin lesions included topical and systemic antibiotic, antifungals, and topical steroids with little effect. In two patients, lesion completely resolved upon chemotherapy, which was part of HSCT conditioning or lymphoma treatment (Figure 1B).

### Oncological Manifestations

Twenty of our 35 NBS patients (57%) developed oncological complications, with the median age of onset 6.0 years (2–18 years) (Table 1). Four patients consecutively developed two malignancies.

Majority of patients had tumors of lymphoid origin: 11 mature B cell lymphomas [diffuse large B-cell lymphoma (DLBCL)-10, Burrkit’s lymphoma-1], 4 T cell lymphomas (2 lymphoblastic, 2 peripheral not otherwise specified), 7 T and B lymphoblastic leukemias and 1 patient had T-prolymphocytic leukemia. Patients with mature B-cell lymphomas were treated according to modified BFM-NHL 2010 core protocol chemotherapy and rituximab (13), peripheral T-cell lymphomas were treated according to individual schemes, leukemias were treated according to approved MB-ALL2008 or ALL-BFM-2005 protocols. A reduction of doses was implemented in seven patients, the majority (13 patients) received non-reduced chemotherapy with no significant toxicity. Two of the patients were irradiated (prior to NBS diagnosis), with no unexpected toxicity or second tumor development. Relapse of lymphomas occurred in two patients (one in DLBCL without HSCT and one in T cell lymphoma after HSCT), no leukemia relapses were noted. One patient developed a solid tumor—ganglioglioma, she received palliative treatment only and eventually died. Overall tumor progression was a cause of death in five patients.

Lymphomatoid granulomatosis has been diagnosed in three patients (two grade II and one grade III), it was successfully treated in all.

### Laboratory Data and TREC/KREC Assay

T, B lymphocytes subsets and immunoglobulins levels were assayed in all patients. Most patients (28 patients) had laboratory signs of immunodeficiency. Twenty four out of 35 patients had T lymphocyte concentration below 1,000 cells/μl, 20 had B lymphocyte concentration below 200 cell/μl. Significant decrease (more than two SDs from mean) of IgG only was noted in five patients, two or more immunoglobulin classes—in 17.

**Figure 2:**
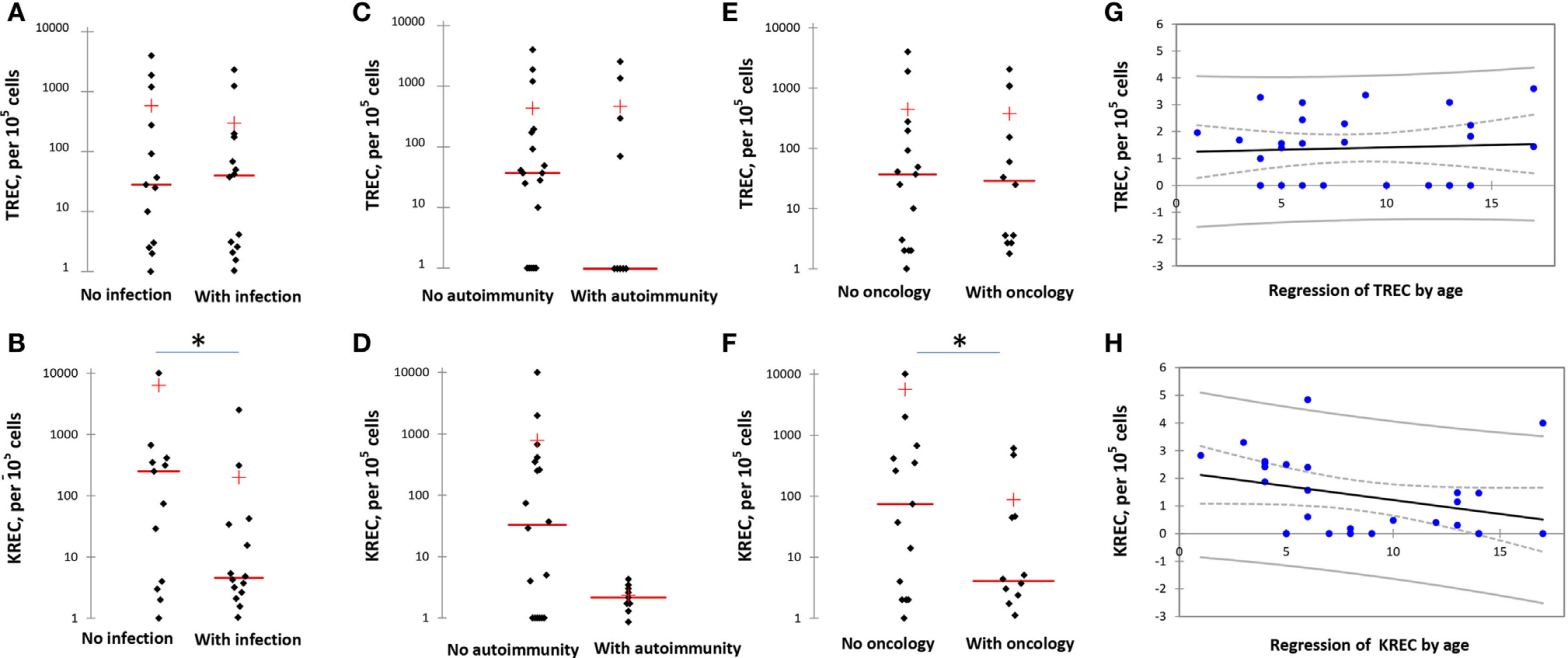
T-cell excision circle (TREC) and kappa-deleting recombination excision circle (KREC) levels in various groups of Nijmegen Breakage Syndrome (NBS) patients. **(A,B)** TREC and KREC levels in NBS patients with and without infection. **(C,D)** TREC and KREC levels in NBS patients with and without autoimmune complications. **(E,F)** TREC and KREC levels in NBS patients with and without malignancies. **(G,H)** Age-dependent distribution of TREC and KREC levels in NBS patients. Asterisk indicates statistically significant difference between the groups. TREC/KREC levels are expressed per 10^5^ cells. Note logarithmical scale of the graphs.

Twenty eight patients had TRECs/KRECs assayed. Twenty two patients had low (below 1,000/10^5^ cells) TREC and 24 had low (below 1,000/10^5^ cells) KREC numbers (Figure 2). When groups of NBS patients with or without severe infectious episodes were compared, TREC numbers were not different between the groups (mean 293.2 ± 178 vs 581.1 ± 301, *p* = 0.8). Yet, KREC numbers were much lower in the group with severe infections (168.4 ± 143 vs 6,316.3 ± 4,955, *p* = 0.07). The same trend was noted when patients with or without autoimmune complications were compared: there was no difference in TREC numbers between the groups (430.7 ± 192 vs 431.4 ± 329, *p* = 0.6), but KREC levels were lower in the group with autoimmunity, though the difference did not reach statistical significance (2.7 ± 0.4 vs 782 ± 546, *p* = 0.15). In the groups with and without oncological complications, KREC levels were lower in the first group (53.5 ± 27 vs 5,588.3 ± 5,142, *p* = 0.05), TREC levels were not different (420 ± 187 vs 440 ± 312.6, *p* = 0.7) (Figure 2).

When analyzed in the context of patients’ age, TREC levels varied independent of it, whereas KREC numbers decreased with age (Figure 2).

### Hematopoietic Stem Cell Transplantation

Fifteen patients received HSCT. According to the Institutional protocol, indications for HSCT were frequent and severe infections in 5, oncological complications in 8, lymphomatoid granulomatosis in 2. All patients with maligancies were in complete remission of their oncological disease at the time of the HSCT, median time of remission before HSCT was 20.8 months (2.4–52.5). Four patients were transplanted from HLA matched (10/10, *n* = 3; 9/10, *n* = 1) siblings, 11 from HLA matched unrelated donors (10/10, *n* = 10; 9/10, *n* = 1) The median follow-up period was 8.5 months after transplantation (1.0–43.0 months). None of the 15 patients developed GVHD 2–4 grade and severe visceral toxicity after conditioning. Three patients rejected graft (median 6.0 months after HSCT; range 2.0–10.7); one of them died 17 months after transplantation from newly developed high-grade lymphoma. One patient died from relapse of lymphoma 2.7 months after HSCT, and one more patient died of fulminant ADV hepatitis. Currently, 12 (81.3%) of 15 patients are alive, 10 (62.5%) of them in good clinical condition and with a functioning graft. The two remaining patients with graft rejection are being prepared for the second HSCT.

### Survival

Twenty-seven patients (74%) were alive at the time of analysis, with a median age of surviving patients 10 years (from 1 to 19). In the group of patients who underwent HSCT, 12 out of 15 were alive. Causes of death were fulminant ADV hepatitis in one and malignancies in the other two: one patient had relapse of T cell lymphoma on day +60 after HSCT, one rejected graft 11 months after HSCT and simultaneously developed high-grade fatal T cell lymphoma.

**Figure 3:**
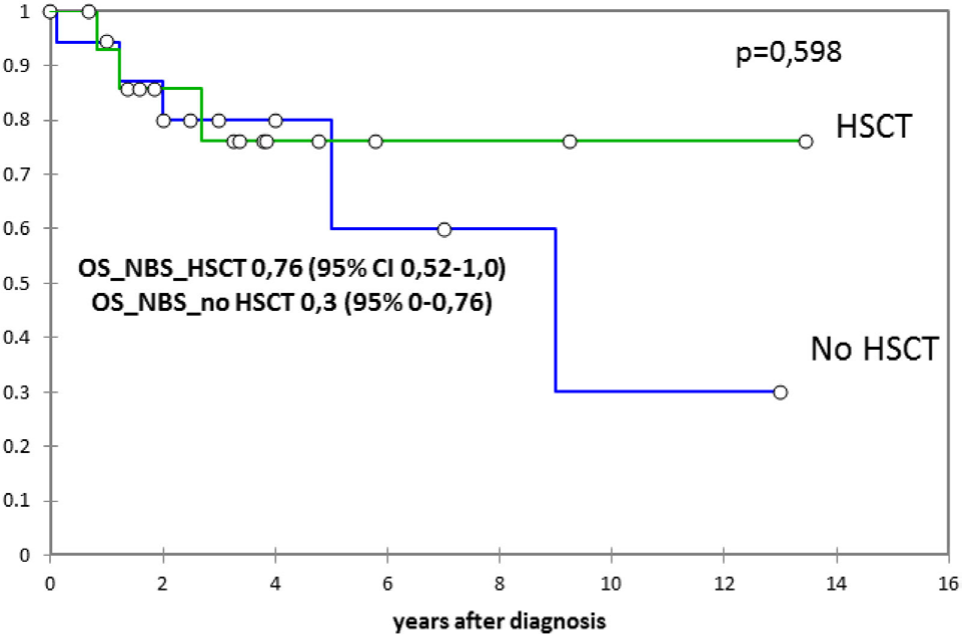
The probability of the overall survival since Nijmegen breakage syndrome (NBS) diagnosis in 15 patients who underwent hematopoietic stem cell transplantation (HSCT) and in 20 non-transplanted patients (expressed as percentage ± SD). Probability of overall survival was estimated by the Kaplan-Meier product limit and were expressed as percentage ± standard deviation. The two-sided log-rank test was used for comparisons.

From the time of NBS diagnosis, probability of overall survival in this group was 0.76 (follow-up 0.5–14 years) (Figure 3).

In non-transplanted patients, 15 out of 20 are alive. Causes of death in the deceased non-transplanted patients were sepsis in one, sepsis during lymphoma treatment in one, and tumor progression in three. From the time of NBS diagnosis, probability of overall survival in this group was 0.3 (follow-up 0.3–13 years) (Figure 3).

## Discussion

Nijmegen breakage syndrome is a unique primary immunodeficiency that has very uneven distribution in the world, with highest prevalence in Eastern European countries. Several cohorts from Poland and other eastern European countries have been described (14, 15). The largest and recent study by Wolska-Kuśnierz et al. contained retrospective analysis of 149 NBS patients registered in the ESID database (6). Our prospective study reports 35 predominantly pediatric patients followed in one center, which provides the advantage of a uniform approach to laboratory investigations and treatment. Both studies have similar demographics of the patients’ cohorts (Table 2).

**Table 2:** Comparison of clinical and laboratory findings in two studies of Nijmegen Breakage Syndrome patients: current (Deripapa et al.) and Wolska-Kuśnierz et al. (6).

Categories	Current study by Deripapa et al.	Wolska-Kuśnier et al. (6)
Number of patients	35	149
M/F ratio, %	46/54	51/49
Median age, years	13.4	14.3
**Infections**		
Mild	49	34
Severe	51	66
Opportunistic infections, %	0	5
Immunoglobulin substitution, %	100	68
Correlation of T cell defect with severity of infections	No correlation of infections and T-cell excision circles	No correlation of infections and CD3+ lymphocyte numbers
Laboratory predictors of severe infections	Low kappa-deleting recombination excision circles (KRECs)	Low Igg
Autoimmune complications, %	34	10
Interstitial lymphocytic lung disease, %	11	No/not reported
Malignancies, %	57	42
Median age of first malignancy	6.0	10.3
Solid tumors, % of all malignancies	4	11
Died from malignancy, %	25	44
Reduced chemotherapy protocols, %	35	“Mostly reduced”
Underwent hematopoietic stem cell transplantation (HSCT), %	43	10
HSCT age		8.8
Alive after HSCT, %	80	71
Relapse of malignancy after HSCT, %	7	0
Deceased patients, %	26	39
Median age of death, years	9.5	11.1
**Cause of death**		
Malignancy and complications, %	75	59
Infections, %	12.5	14
Infections during HSCT, %	12.5	5
Other, %		22
Poor prognosis predictors, clinical	No HSCT	Severe infections malignancy
Poor prognosis predictors, laboratory	Low KRECs	Not found

As expected, most of our patients were Russians or Ukranians. Interestingly, one girl comes from consanguineous gypsy family living in Russia. NBS is one of the most frequent combined primary immunodeficiencies in Russia, and considering typical syndromic features, it should be readily recognized in infancy. However, the average delay of the NBS diagnosis was 6.5 years. Most patients were diagnosed by hematologist/oncologist at the time of oncological manifestation, and in some cases even later.

The main causes of morbidity in our study group were oncological and autoimmune complications. Among the latter, there were four patients with ILLD—recently recognized PID complication (16, 17), which has not been described in NBS to date. ILLD in PID often mimics interstitial and/or fungal pneumonia, the diagnosis requires high level of awareness and lung biopsy, which, to our knowledge, is not easily available in some clinics. Therefore, we think that lack of ILLD in patients in Wolska-Kuśnierz et al. study reflects hypodiagnostics of this debilitating condition.

Interestingly, in contrast to other PIDs, where immune cytopenias are the main autoimmune complication, they were observed only in 0% (our study) to 3.6% (Wolska-Kuśnierz et al.) of NBS patients.

Previously, skin sarcoid-like granulomatous lesions have been reported in two patients with NBS (18, 19), as well as in other PIDs, with large predominance of patients with ataxia-telangiectasia (AT) (20–22). Not surprisingly, in our group of patients with another DNA repair defect, five NBS patients developed granulomas. The RA27/3 vaccine strain of rubella virus has been found in some of PID granulomas before (21–23). We detected the rubella virus in homogenized granuloma tissue samples from three out of five of our patients. In our experience and according to other case reports theses, granulomas are quite resistant to various modes of therapy and in our patients have resolved only after intensive chemotherapy conducted as part of the tumor treatment or HSCT conditioning. Interferon-alpha-2b therapy has been shown to be effective in the treatment of hypertrophic herpes-associated skin lesions in DOCK8-deficient patients (24). It would be interesting to use a similar approach in skin granulomas in NBS.

The high prevalence of malignancies is a known feature of DNA repair genetic defects. Wolska-Kuśnierz et al. report 42% frequency of tumors in NBS, with 44% mortality (6). We observed an even higher frequency of oncological complications (57%), yet, the mortality rate was much lower—25% (Table 2). This might be partially explained by the fact that our patients were mostly under 18 years of age, and pediatric tumors frequently have better prognosis. Yet, in our opinion, the main contributing factor to the better survival rate in our patients’ cohort with lymphoid tumors was the delivery of full-dose chemotherapy according to appropriate protocols (along with IVIG treatment), despite the theoretically elevated hazard of intolerable toxicity in chromosome-repair defect syndromes (9, 25).

The majority of studies demonstrating chemotherapy-associated toxicity in patients with DNA repair defects have involved predominantly AT patients or mixed AT and NBS groups (9, 26, 27). It has been shown that NBS patients carrying the c.657_661del5 mutation have residual NBN activity due to the function of two truncated proteins—p26 and p70 (28), which makes DNA repair in NBS less affected that in AT. In most of our patients (65%), conventional unmodified regimens were used and were well tolerated, suggesting that immediate tolerance of chemotherapy is not necessarily affected in NBS patients at least in Slavic population. Considering the high rate of tumor relapses in NBS, the actual tolerability of standard chemotherapy protocols and correlation with secondary tumor occurrences require further investigation in large and homogenous groups of NBS patients.

The combined nature of immunodeficiency in NBS has been described in details before. In our cohort, 22 patients (62%) had decreased levels of one or more classes of immunoglobulins, 20 (57%) had low B lymphocytes, and 24 patients (68%) had low T lymphocytes subsets. Even though many of our patients were started on immunoglobulin and antimicrobial prophylaxis during late childhood due to a delayed diagnosis, they all received IVIG at the time of the study, irrespective of IgG levels. This fact might explain a somewhat lower rate of severe and lethal infections in our cohort by comparison with that of Wolska-Kuśnierz et al. (Table 2), and the fact that we did not register opportunistic or fungal infections in our patients. Since NBS is a combined immunodeficiency, we postulate that most (if not all) patients require IVIG substitution, and this decision should be made based on antibody-specific responses.

T-cell excision circles and KRECs evaluation has become a widely used assay for PID screening and follow-up. Low TREC and KREC numbers have been demonstrated in several NBS patients before (7, 8). Since TRECs are known to correlate with naïve and not peripherally expanded T lymphocytes numbers, they are better predictors of immunodeficiency severity that total T cell numbers. We studied TREC and KREC numbers in a subgroup of 28 NBS patients and found that 78% had TRECs and 85% had KRECs below cutoff level (Figure 2). This reflects a higher proportion of patients with abnormal T and B immunity compartment than when lymphocyte phenotyping and immunoglobulin levels were used as a marker of immunodeficiency. These findings also provide a background for possible NBS detection by neonatal screening.

Low KREC numbers (in contrast to TREC numbers) seem to be predictors of severe infections, oncological, and possibly autoimmune complications (Figure 2) and, therefore, predictors of severe course of the disease. Interestingly, though TREC levels varied in patients independent of age, KREC levels progressively decreased with age (Figure 2) in opposite to normal subjects in whom KRECs stay around the same level through life. Of note, even though Wolska-Kuśnierz et al. did not study TRECs/KRECs, they also demonstrated that disease severity in NBS correlates with B, but not T cell defect (6). Overall, these findings confirm the significant role of B cell defect in NBS pathogenesis and suggest that, in NBS, KREC numbers are reliable predictors of disease severity.

Previously, HSCT in NBS was considered dangerous due to risks of conditioning-related toxicity and the fact that immune reconstitution is not able to fully correct malignant predisposition in this group of patients. Hence, there are only few reports of successful HSCT in NBS (29–32), describing less than the total of 30 patients transplanted worldwide. The reported HSCT survival in them is above 70%.

Of 15 NBS patients who underwent HSCT in our center to date, 12 are alive and 10 have good chimerism and immune reconstitution. According to our current Institutional protocol, only NBS patients with life-threatening complications (malignancies, severe infections, etc.) are eligible for HSCT. In our study, one out of three deceased patients in HSCT group died of tumor relapse. When probability of survival since NBS diagnosis (when presumably a decision about HSCT is made) was analyzed in our group, it was much more favorable in the group of transplanted patients (Figure 3). Since HSCT in NBS should reduce, but not completely cure, predisposition to malignancies, longer post-transplant follow-up is needed. However, the success of HSCT in NBS reported by us and others raises the question of indications for stem cell transplantation in NBS before malignancy or severe infection occurrence.

In conclusion, our prospective study demonstrates high rate of non-infectious complications in NBS. We provide preliminary information that TREC and especially KREC levels can be used as predictors of these complications, as well as a tool for neonatal screening of NBS. HSCT is a viable treatment option in NBS and should be especially considered in patients with low TREC/KREC numbers early on, before they develop life-threatening complications.

## Ethics Statement

This study was carried out in accordance with the institutional guidelines with written informed consent from all subjects/their parents. The protocol was approved by the Dmitry Rogachev National Research Center of Pediatric Hematology, Oncology and Immunology Ethics Committee.

## Author Contributions

ED was responsible for the design of the study, patient recruitment and data acquisition, analysis, interpretation, and drafting of the manuscript. YR and AL were involved in the study design, interpretation, and drafting of the manuscript. DB and LS were involved in HSCT protocol and data analyses, helped revise the manuscript, and approved the final version of the manuscript. NM was involved in malignancies treatment protocol and data analyses, helped revise the manuscript, and approved the final version of the manuscript. ND, MG, DA, and GP were involved in analysis of laboratory data, were involved in revising the manuscript, and approved the final version. AP, MM, AM, and AS involved in study design, revising the manuscript, and approved the final version.

## Conflict of Interest Statement

The authors declare that the research was conducted in the absence of any commercial or financial relationships that could be construed as a potential conflict of interest.
